# Evaluation of Foramen Magnum for Sex Determination among the Population of Dakshina Kannada District: A Retrospective CBCT Study

**DOI:** 10.1155/2024/6825489

**Published:** 2024-08-23

**Authors:** Junaid Ahmed, Namrata, Nanditha Sujir, Nandita Shenoy, Archana M., Srikant Natarajan

**Affiliations:** ^1^ Department of Oral Medicine and Radiology Manipal College of Dental Sciences Mangalore Manipal Academy of Higher Education, Manipal, Karnataka 576104, India; ^2^ Department of Oral Pathology and Microbiology Manipal College of Dental Sciences Mangalore Manipal Academy of Higher Education, Manipal, Karnataka 576104, India

## Abstract

**Background:**

This study aims to evaluate the accuracy rate of foramen magnum dimensions in determining sex among the South Indian population using discriminant functional analysis.

**Methods:**

An observational study in which CBCT images from 200 full field of view (FOV) scans were analysed. The dimensions of the foramen magnum were measured. Intra- and interobserver reliability were calculated. Independent *t*-tests were used to compare the various parameters between sexes. Stepwise discriminant function analysis was used to determine sex.

**Results:**

A total of 200 CBCT scans were included in the study. The mean age (±SD) was 25.66 (±7.11) years among males and 24.64 (±5.12) years among females. The measurements and the circumference of the foramen magnum were significantly (*p* < 0.001) greater in males than in females. The univariate analysis of foramen magnum measurements reached an accuracy rate of 73.5% in sex determination. The discriminant function analysis combining the foramen magnum measurements and circumference yielded an overall predictability rate of 66.5% for determining sex.

**Conclusion:**

Taking into account the predictability rate of sex based on foramen measurement in the present population, it can be concluded that its applicability should be limited to cases associated with fragmentary skull bases.

## 1. Introduction

Determining the biological profile of unidentified human remains is one of the most challenging aspects of forensic science, and sex determination plays a crucial role in reconstructing the biological profile of an unknown individual [1, 2]. Sex determination can be an accurate process when the complete set of skeletal remains is available and the bones display the regular anatomic features that indicate sexual dimorphism. However, atypical features with anatomic variations and fragmentation of skeletal remains due to natural disasters, explosions, and other mass disasters can result in skeletal features that make it difficult to categorize the remains as male or female. In this context, anthropometric analysis of skeletal structures can be utilized for sex determination [3]. In the craniofacial region, measurements of the gonial angle, ramus length and width, and bony and bicondylar measurements [4–6] and measurements of the length, height, circumference of the head, circumference of the occipital condyles, and foramen magnum (FM) are known to be sexually dimorphic [7–12].

FM is a three-dimensional opening in the central region of the base of the occipital bone and is an anatomical landmark that marks the transition zone between the spine and the skull. The basicranium with the foramen magnum and the area around it are relatively indestructible due to the nature of the hard tissue, with the thickness of the occipital bone and its protective anatomical position making it amenable for use in forensic identification and sex determination [13, 14]. Hence, FM provides an accessible alternative to invasive methods of examination, such as DNA analysis, and thus has become a recent focus in many forensic investigations [15–17].

Radiographic measurements are a reliable method for examining skeletal structures and are critical for forensic investigations. Three-dimensional imaging, such as computed tomography (CT) and cone beam computed tomography (CBCT), has been increasingly gaining importance for forensic investigations, as these imaging modalities allow for accurate visualization of skeletal structures, especially in comparison to two-dimensional imaging [18]. CBCT is an accurate alternative to CT for skeletal imaging of the head and neck, as it can be acquired with a significant reduction in cost and radiation exposure [19]. There are few studies in literature that have evaluated the utility of foramen magnum of sex determination. However, there are no reported studies that provide morphometric data for the population of Dakshina Kannada district. The objective of the present study was to evaluate the accuracy of foramen magnum measurements using CBCT images in determining an individual's sex among the population of Dakshina Kannada district.

## 2. Materials and Methods

This comparative study was conducted after institutional ethics committee approval was obtained (Protocol no 19128). Based on an article by Gargi et al. [13] published in the year 2018, the standard deviation (SD) values of the two groups were 1.79 (*Z*_1_) and 1.91 (*Z*_2_) at an alpha (*α*) error of 3% power (*β*) of 95%, and with an average standard deviation of 1.8 (*σ*), using the formula *N*=(2(*Z*_1−(*α*/2)_+*Z*_1−*β*_)^2^*σ*^2^)/*d*^2^, a sample size of 100 per group was calculated. Hence, a total of 200 scans were included, of which 100 were from males and 100 were from females. Full field of view (FOV) CBCT scans were retrieved from the department archives. Full-FOV scans of patients >20 years of age were included in the study. The exclusion criteria were (a) scans of patients who were <20 years of age, (b) a history of craniofacial surgery, (c) scans of patients with pathologies such as fractures and tumors, (d) scans of patients with maxillofacial deformity, and (e) images with distortions/artifacts. A Planmeca ProMax 3D Mid CBCT machine (Romexis Version 4.6.2 R; Planmeca, Helsinki, Finland) was utilized for the full-FOV 3D scans with a voxel size of 0.6 mm and exposure parameters set at 90 kV, 5.6 mA, and an 18 sec exposure time. The multiplanar reconstructed images were aligned in three planes such that the axial orientation was aligned to the Frankfort horizontal plane, the coronal orientation was aligned to the plane along the anterior margins of the right and left external acoustic meatus, and the sagittal orientation was aligned to the midsagittal plane as described by Balachandran et al. [20].

**Figure 1:**
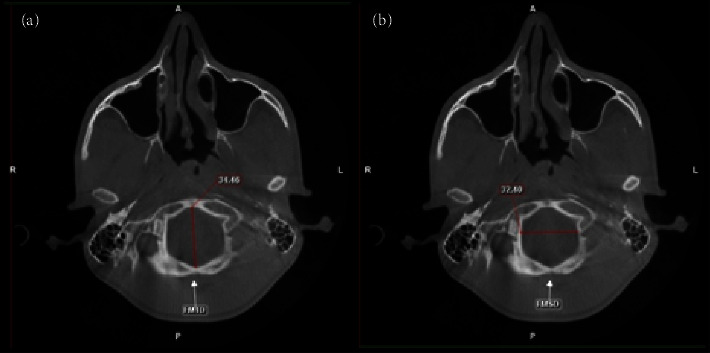
CBCT scan showing measurements of the foramen magna. (a) Foramen magnum sagittal diameter; (b) foramen magnum transverse diameter. Picture source: Department of Oral Medicine and Radiology, MCODS, Mangalore.

The measurements were carried out on axial and coronal sections (Figure 1). Axial sections were used to measure the sagittal and transverse circumferences of the FM. The sagittal diameter (FMSD), transverse diameter (FMTD), and circumference of the FM were analysed (FMC) (Figure 1). The FMSD was recorded as the largest anteroposterior dimension of the FM, the FMTD was recorded (Figure 1) as the largest FM width, and the FMC was calculated using the formula—2 × 3.14 × FMTD/2 (circumference formula). All measurements were carried out by two experienced maxillofacial radiologists with over 5 years of experience at different time intervals. The radiologists were unaware of the sex of the patient. The principal investigator repeated the measurements of 20 scans after 2 months. Each of the scans was number-coded, and the measurements of the scans were correlated to the sex of the individual by another investigator. Intra- and interobserver reliabilities were calculated.

Statistical analysis: Statistical analysis of the collected data was performed using SPSS version 20.0 (IBM SPSS® Statistics). The intraclass correlation coefficient was used to determine the inter and intraobserver reliability. A comparison of the difference between males and females among the examined dimensions of FM was performed using the independent *t*-test. Equations predicting sex differences using the examined parameters were derived via discriminant analysis. Additionally, the receiver operating characteristic (ROC) curve, AUM, sensitivity, and specificity were also estimated. *p* value <0.5 was considered significant.

## 3. Results

A total of 200 CBCT scans were included in the study. The mean age (±SD) among males was 25.66 (±7.11) and that among females was 24.64 (±5.12). The intraclass correlation values ranged from 0.7 to 0.8 for intraobserver reliability and 0.6–0.7 for interobserver reliability, which indicates good intraobserver and moderate interobserver reliability.  Table 1 includes the mean values of the various measurements, with all the parameters showing statistically significant differences between males and females.

**Figure 2:**
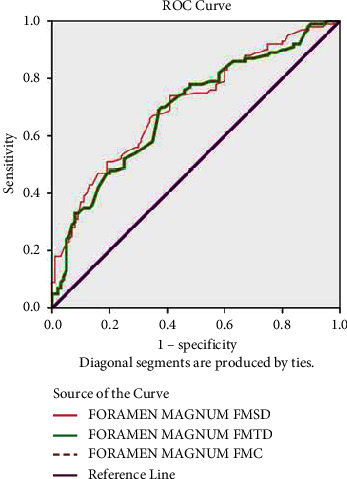
ROC curves for various parameters.

Figure 2 shows the ROC curve for the various parameters, with FMSD showing the greatest accuracy in sex identification. The area under the curve (AUC) showed that the FMSD had acceptable accuracy (Table 2). The equation derived through discriminant function analysis for sex determination is summarized in Table 3. The highest accuracy of 73.5% was noted for FMSD and FMTD. The sensitivity and specificity of each cut-off value were calculated and are summarized in Table 4.

## 4. Discussion

Anthropometric studies related to FM have been carried out through direct measurements, 2-dimensional radiographs, and 3-dimensional imaging modalities such as CT and CBCT. Catalina-Herrera [21], Uysal et al. [22], Uthman et al. [23], Radhakrishna et al. [24], Sk et al. [25], and Patel and Mehta [26] reported that FM shows sexual dimorphism. In contrast, studies by Kanchan et al. [15] and Shepur et al. [27] have shown that FM does not show any sexual dimorphism. In our study, we observed significant differences in the parameters of FM between sexes. The length of FM develops fully by the 5th year of life, and the growth in breadth progresses until the end of the first decade. As no significant changes occurred thereafter, age was considered to be noncontributory for the development of a sex determination model for this study [28].

Radhakrishna et al. [24] analysed FM for sex differences using standard osteometric techniques and found that anteroposterior and transverse diameters were greater among male skulls. A similar study was performed by Edwards et al. [29] and Raghavendra Babu et al. [30], where an additional parameter, i.e., the area of FM, was explored for sexual dimorphism. Their results were similar to those of the study conducted by Radhakrishna et al. [24], who noted that the dimensions were significantly greater in males. Our study also revealed similar results, with the dimensions being greater in males. The dimensions of the various parameters were similar to those of Gargi et al. [13], who conducted a study among the North Indian population, and Mustafi et al., who conducted a study among the population of Eastern India [31]. Abo El-Atta et al. [32] noted variations in the measurement of FM among different ethnic groups and concluded that the ethnicity of the subject undergoing analysis should be taken into account for sex determination using FM. Bahşi et al. [33] Akay et al. [34] and Evli et al. [35] evaluated linear measurements of FM length and width in CBCT scans among the population in Turkey and found significant differences in the measurements between males and females. The values of length and width among males and females were comparable to those in our study.

The predictability of FM measurements in differentiating sex was 65.4% for the transverse diameter and 86.5% for the anteroposterior diameter [30]. Edwards et al. [29] concluded that the accuracy of all variables was 66% according to discriminant function analysis, and binary logistic regression was comparatively poor at classifying females despite an overall classification rate of 66.4%. Jaitley et al. [2] used CBCT to evaluate the dimensions of FM and found that the FM area was the best discriminant parameter for studying sexual dimorphism, with an overall accuracy of 72%. In our study, the sagittal and transverse diameters of the FM showed an overall accuracy of 73.5%, which is greater than that reported in other studies. Tambawala et al. [1] analysed the maximum length and width of the FM using CBCT imaging, and the overall accuracy rate for sex determination was found to be 66.4%. In a similar study performed using 70 CBCT scans, Kotha et al. [36] reported that the overall accuracy of sex determination was 67.1%. Gargi et al. [13], in a similar study, reported an accuracy of 90.9%. Kartal et al. [37] found that sex could be determined with an accuracy rate of up to 88.2% using FM measurements in CT scans. In a similar study, Abo El-Atta et al. [32] utilized CT for the analysis of FM, including the analysis of structures around the FM, such as the occipital condyle, and found greater accuracy in the determination of sex using measurements of multiple anatomic structures. Kartal et al. achieved an accuracy of 84.6% using measurements of FM and discriminant function analysis by adding additional data from other Turkish studies, emphasizing the need for larger samples [38]. Similar studies performed in Saudi Arabia using CT images have yielded an accuracy of 52%–65% using discriminant function analysis [17, 38]. Ilguy et al. [39] reported a higher accuracy rate of 83.2% in their CBCT study, which analysed mandibular parameters along with FM. According to Ramamoorthy et al. [40], increasing the number of variables for sex determination by discriminant function analysis improves the percentage of accurate sex identification. However, in our study, the combination of FM parameters reduced the accuracy of sex determination. It could thus be recommended to utilize additional anatomic structures such as the occipital condyle or mandible (intercondylar and intergonial distances) along with the FM to improve accuracy, as observed in other studies. The variation in accuracy across various studies may be due to variable sample sizes, methodological differences, and variations in the statistical tests adopted. The accuracy of sex determination with parameters of FM may be improved by utilizing additional parameters. Further studies with a larger sample size are essential to determine similar results.

FM is easily accessible to visual examination, and manual measurements can be done directly. Manual measurements can be time consuming and require a trained specialist. When the number of cases is small, the feasibility is seldom an issue. However, considering mass disaster and requirement of identification of high number of skeletal remains, imaging can be advantageous. In the era of artificial intelligence (AI), imaging provides for automation of measurements and can help save time and resources. Although the focus of this study was not AI, validation of the process of image acquisition and measurements would be a prerequisite to develop AI models in the future. Thus, this study can be a foundation for future studies that can use artificial neural networks in sex determination.

Among the newer imaging modalities, CBCT images provide versatile analysis of craniofacial structures, which had not been possible with conventional 2D imaging. Our study demonstrated moderate interobserver and good intraobserver reliability, indicating reproducibility. Linear morphometric measurements using CBCT have proven to be accurate, and the results can be compared to those of previous studies in the literature, which were performed through direct skull measurements or CT. Additionally, in comparison to CT, CBCT offers the advantages of reduced scan time, reduced cost, and reduced radiation dose, resulting in an operational advantage. To date, four Indian studies have evaluated linear measurements of foramen magnum using CBCT in the West Bengal, Uttar Pradesh, Andhra Pradesh, and Maharashtra states [1]. However, discriminant function analysis was used only in the studies conducted by Tambawala et al. [1] and Kotha et al. [36]. In the present study, we considered the vast geographic and ethnic variations in India, and our study focused on the South Indian population in the state of Karnataka. Additionally, our study also evaluated the sensitivity, specificity, and AUC for mean values related to foramen magnum, which significantly contributes to the applicability of the methods [38]. The limitation of the present study is that the results apply to the population of Dakshina Kannada district and cannot be generalized. Also, repeated CBCT measurements to determine intraobserver reliability were carried out after 2 months, which could ideally be executed within 1-2 weeks. A multicentric study with sampling techniques to reflect the population of various states of India is desirable for a broader applicability of foramen magnum measurements for sex determination. Considering the predictability rate of sex based on foramen measurement in the present population, it can be concluded that its applicability should be limited to cases associated with fragmentary skull bases.

## 5. Conclusions

The present study highlights the reliability of using CBCT in craniometric analysis in forensic dentistry. Our findings support the sexual dimorphism of FM, with statistically significant differences in the length and width of FM among the sexes. The accuracy of sex determination using discriminant function analysis was found to be moderate (66.5%). Therefore, it can be used as an adjunct for sex determination along with other conventional methods. The role of CBCT in morphometric measurements of various craniofacial structures has multiple possibilities in the field of forensic radiology and should be explored further.

## Figures and Tables

**Table 1:** Comparison of various parameters between males and females.

Parameters	Male (*n* = 100)	Female (*n* = 100)	*t*	*p* value
	Mean ± SD	Mean ± SD		
FMSD	36.65 ± 2.7	34.69 ± 2.26	5.552	<0.001^∗^
FMTD	31.87 ± 2.62	30.07 ± 2.4	5.045	<0.001^∗^
FMC	100.06 ± 8.21	94.43 ± 7.55	5.045	<0.001^∗^

FMSD: foramen magnum sagittal diameter (mm), FMTD: foramen magnum transverse diameter (mm), FMC: foramen magnum circumference, and SD: standard deviation. Statistical test: independent *t*-test B. ^∗^A statistically significant difference.

**Table 2:** The area under the curve of various parameters.

Area under the curve					
Variables	Area	Std. Error	*p* value	Asymptotic 95% confidence interval	
				Lower bound	Upper bound
FMSD	0.707	0.036	<0.001^∗^	0.636	0.778
FMTD	0.692	0.037	<0.001^∗^	0.619	0.765
FMC	0.692	0.037	<0.001^∗^	0.619	0.765

FMSD: foramen magnum sagittal diameter (mm), FMTD: foramen magnum transverse diameter (mm), FMC: foramen magnum circumference, and SD: standard deviation. Statistical test: independent *t*-test B. ^∗^A statistically significant difference.

**Table 3:** Equations derived through discriminant function analysis for determining sex.

Parameter	Equation	Percentage of males correctly classified	Percentage of females correctly classified	Overall accuracy (%)	Male centroid	Female centroid	Demarcating point
FMSD	Discriminant function (D) = −14.349 + (0.402) × (FORAMEN MAGNUM FMSD)	65	66	73.50	0.393	−0.393	35.69403
FMTD	Discriminant function (D) = −12.329 + (0.398) × (FORAMEN MAGNUM FMTD)	68	63	73.50	0.357	−0.357	30.97739
FMC	Discriminant function (D) = −12.329 + (0.127) × (FORAMEN MAGNUM FMC)	68	63	59.20	0.357	−0.357	97.07874
Combined analysis for foramen magnum parameters	Discriminant function (D) = −16.003 + 0.266 (FMSD) + 0.210 (FMTD)	64	69	66.5	0.448	−0.448	NA

The sectioning point was zero for all the parameters. FMSD: foramen magnum sagittal diameter (mm), FMTD: foramen magnum transverse diameter (mm), and FMC foramen magnum circumference.

**Table 4:** Sensitivity and specificity of various parameters for identifying the discriminating point.

Parameters	Sensitivity (%)	Specificity (%)
FMSD	66	66
FMTD	69	63
FMC	70	62

FMSD: foramen magnum sagittal diameter (mm), FMTD: foramen magnum transverse diameter (mm), FMC: foramen magnum circumference.

## Data Availability

The datasets generated and/or analysed during the current study are not publicly available due to institutional policy but are available from the corresponding author upon reasonable request.
